# Stress of working abroad: a systematic review

**DOI:** 10.1007/s00420-018-1333-4

**Published:** 2018-07-02

**Authors:** Shotaro Doki, Sinichiro Sasahara, Ichiyo Matsuzaki

**Affiliations:** 1 Institute of Psychiatry, Psychology and Neuroscience, King’s College London, London, UK; 2 Faculty of Medicine, University of Tsukuba, Tsukuba, Japan; 3 International Institute for Integrative Sleep Medicine, University of Tsukuba, Tsukuba, Japan

**Keywords:** Acculturation, Business travellers, Expatriates, Immigrants, International assignment

## Abstract

**Purpose:**

Many companies target international markets to expand their business. Companies need to manage international teams with a wide variety of skills, knowledge and values to run their business effectively; however, there are many issues of acculturation stress. Not only business travellers and expatriates, but also immigrant workers have issues adjusting to foreign culture. The aim of the present study is to identify the stress factors affecting foreign-born workers via a systematic review.

**Methods:**

The systematic review was conducted using PubMed, PsycINFO, Embase and Cochrane Library databases. Articles on the subject of workers living abroad, such as immigrants, expatriates and business travellers, were included. The risk of bias in the included articles was assessed using the Cochrane Collaboration’s tool for assessing risk of bias for randomised controlled trials (RCTs), RoBANS for non-RCT studies, and CASP for qualitative studies.

**Results:**

For the systematic review, 45 out of 14,994 articles were analysed. Six components, communication, cultural differences in the workplace, daily life, relationships with family and colleagues, financial problems and social inequality, were extracted.

**Conclusion:**

Foreign-born workers are suffering from acculturation and occupational stress. The results of the present study can help greatly with understanding of the stress structure of working abroad.

**Electronic supplementary material:**

The online version of this article (10.1007/s00420-018-1333-4) contains supplementary material, which is available to authorized users.

## Introduction

In recent times, many professional people have travelled abroad to work; for example, the number of business visits to the UK was 8.3 million in 2014 (Office for National Statistics 2015). Many companies target international markets; therefore, they send workers to foreign countries. Expanding business is an advantage of globalisation; in contrast, the issue of acculturation for workers has occurred. Premature return from international assignment due to failure to adjust to the foreign culture is a heavy burden for the company because international assignment has higher cost than domestic assignment. Expatriates experience huge cultural differences between their home countries and host countries. Not only expatriates, but also immigrant workers have issues adjusting to foreign culture. They have to greatly change their lifestyle when adjusting to host countries; consequently, many stressful social adjustment problems occur (Cervantes et al. 1991, 2016).

For foreign-born workers, living and working in the host country is more stressful than for native workers. They need to stay in a country which has a different cultural background than they are used to. Working abroad requires adjustment to the host country. There are several pieces of the literature that suggest migrants have less professional support, decreased well-being and experience more mental ill-health compared to native workers (Font et al. 2012; Aalto et al. 2014). Psychological stress can lead to depression and adjustment disorders, which results in suicidal thought and premature return intention (Wang and Takeuchi 2007; Al-Maskari et al. 2011). It is important for foreign workers to elucidate the psychological symptoms and stress factors that affect them when working abroad.

There are many pieces of literature about cross-cultural psychology. According to Lysgaard (1955) and Gullahorn and Gullahorn (1963), there are several steps required for people to adapt to a foreign society. Cross-cultural adjustment is often described as acculturation, which means the processes and consequences of psychological and cultural contact between one culture and another culture (Berry 1997). Berry (1997) categorised acculturation into four types: integration, assimilation, separation, and marginalisation. However, Berry’s theory does not cover all cases of acculturation. Acculturation has many forms which relates to different phenomena and processes. Lazarus (1997) suggested that Berry’s theory was too broad and abstract to describe the framework of acculturation.

There are two studies which have evaluated the adjustment of working abroad. According to Bhaskar-Shrinivas et al. (2005), some paradoxical findings remain and need to be refined. They suggested that the centrality, criticality, and complexity of expatriate adjustment are crucial. Hechanova et al. (2003) suggested that expatriate adjustment is related to job strain, job satisfaction, organisational citizenship, intent to turnover, and job performance. From these studies, the concept of adjusting foreign workplaces has been established. However, how stress and mental disorder are affected by working abroad had not been sufficiently evaluated.

The relationship between acculturation and psychological stress has been controversial (Shen and Takeuchi 2001), and there are three, negative, positive, and curvilinear, relationships between acculturation and psychological stress (Rogler et al. 1991). Acculturation was related to both positive and negative psychological stress directly or indirectly through factors such as social support, personality, and perceived health (Shen and Takeuchi 2001). Mendenhall and Oddou (1985) anecdotally suggested that four dimensions are related to successful international assignments for workers: self-oriented, others-oriented, perceptual, and cultural-toughness. As a theoretical framework, Black et al. (1991) suggested a model of acculturation which has five factors, individual, job, organisational culture, organisational socialisation and non-work, related to the degree of adjustment. Another study that surveyed international assignees suggested that the factors in success are family situation, flexibility and adaptability, job knowledge and motivation, relational skills, and extra-cultural openness (Arthur et al. 1995). Adams and van de Vijver (2015) also reported that expatriates’ stress depends on their cultural distances, support, and purpose, which are also related to the organisational intention for the international assignment. The integration of previous findings is shown in Table 1.

**Table 1:** Four types of factors related to international adjustment or acculturation (based on earlier research)

Theoretical framework			
Individual	Occupational	Support	Cultural
Mendenhall and Oddou (1985)			
Self-oriented	–	Others-oriented	Cultural toughness
Perceptual			
Black et al. (1991)			
Individual	Job	Organisational	Non-work
	Organisational culture	Socialisation	Organisational culture
Arthur et al. (1995)			
Relational skills	Job knowledge	Family situation	Extra-cultural openness
Flexibility	Motivation		
Adaptability			
Adams and van de Vijver (2015)			
Purpose	–	Support	Cultural distances

So far, there are many studies assessing foreign-born workers; however, the subjects or workplaces are limited to specific nationalities or countries. There have been miscellaneous findings since by applying a stressor model acculturation is limited to reactive adjustment to proximal, situational variables neglecting long-term proactive personal (re)development. These studies cannot comprehensively evaluate the theoretical framework of a wide variety of workers and workplaces. The present study aims to assess the factors that deteriorate and mitigate stress amongst workers living abroad by conducting a systematic review. The factors intervening on the relationship between acculturation on the one hand, and stress, and work adjustment on the other hand were searched. Based on the previous cross-cultural adjustment model, to understand the stress structure of working abroad, we focussed on the conceptualisation of acculturation. The hypothesis is that “There are specific factors that either deteriorate or mitigate stress for foreign workers”. This study protocol was registered with PROSPERO (the registration number is: CRD42015029315).

## Methods

### Search

A flow diagram of PRISMA (Preferred Reporting Items for Systematic reviews and Meta-Analyses) recommendations was used to select studies (Liberati et al. 2009). Different databases were searched to determine what kind of stress foreign workers felt and what kind of interventions affected their stress levels. Search terms were selected using a PICO approach, which stands for Population, Intervention, Control and Outcome. We used the search terms “work”, “international”, “abroad” and “immigrant” for Population; “culture” and “adjustment” for Intervention; and “stress” and “mental disorder” for Outcome. Terms for Control were not included deliberately, because the number of controlled studies seemed to be small. The search was conducted on 8th November 2015. The PubMed, PsycINFO, Embase and Cochrane library databases were used to search for articles from 1st January 2000 to 8th November 2015. The search strategy is shown in supplementary A. An expert (SD) selected the articles and two experts (SD and SS) assessed them. First, duplicate articles were deleted from the four databases. Second, the titles/abstracts were assessed, and then relevant articles in full text were collected. Using the inclusion/exclusion criteria following, the full text articles were assessed. Finally, target articles were retrieved. A consensus meeting of three experts (SD, SS, and IM) resolved any disagreements between the two reviewers. We also referred to the bibliographies from retrieved articles.

### Inclusion and exclusion criteria

We included articles whose subjects were workers in all three categories living abroad (immigrants, expatriates and business travellers). In terms of outcomes, we included the studies which were originally aimed at workers’ stress and stress-related illness (e.g. depressive mood, fatigue, exhaustion, burnout, irritation, depression, anxiety and sleep disorder). When there were intervention studies, we included the ones with interventions that offered medication and psychological therapies aimed at reducing stress. We only selected articles written in English.

We excluded studies whose subjects were related to the military and veterans, workers engaged in transportation related to jet-lag (e.g. pilots, cabin attendants, ship crews and astronauts), students, and workers with severe mental illnesses such as psychosis. Articles with a low evidence level such as a conference papers, expert opinions, editorials, letters to the editor and commentary were excluded.

### Study bias and quality assessment

The risk of bias of the included articles was assessed using Cochrane Collaboration’s tool for assessing risk of bias for randomised controlled trials (RCTs) (Higgins and Green 2011). The risk of bias has seven domains: random sequence generation, allocation concealment, blinding of participants and personnel, blinding of outcome assessment, incomplete outcome data, selective reporting and other biases. Each domain was judged as low, high or unclear risk. For non-RCT studies, the risk of bias was assessed by the Risk of Bias Assessment tool for Non-randomized Studies (RoBANS) (Kim et al. 2013). It has six domains: the selection of participants, confounding variables, measurement of exposure, blinding of outcome assessments, incomplete outcome data and selective outcome reporting. Each domain was judged as low, high or unclear risk. For qualitative studies, the quality was assessed using the Critical Appraisal Skills Programme (CASP) (CASP 2013). The checklist has ten questions that help to assess the quality of each study. Each question was answered using “yes”, “no”, or “can’t tell”. When one or two of the first two questions were marked “no” or “can’t tell”, the study was branded as a low-quality study. Finally, a risk of bias table was drawn. The process was conducted independently by two experts (SD and SS). A consensus meeting resolved any disagreements between them.

## Results

**Fig. 1:**
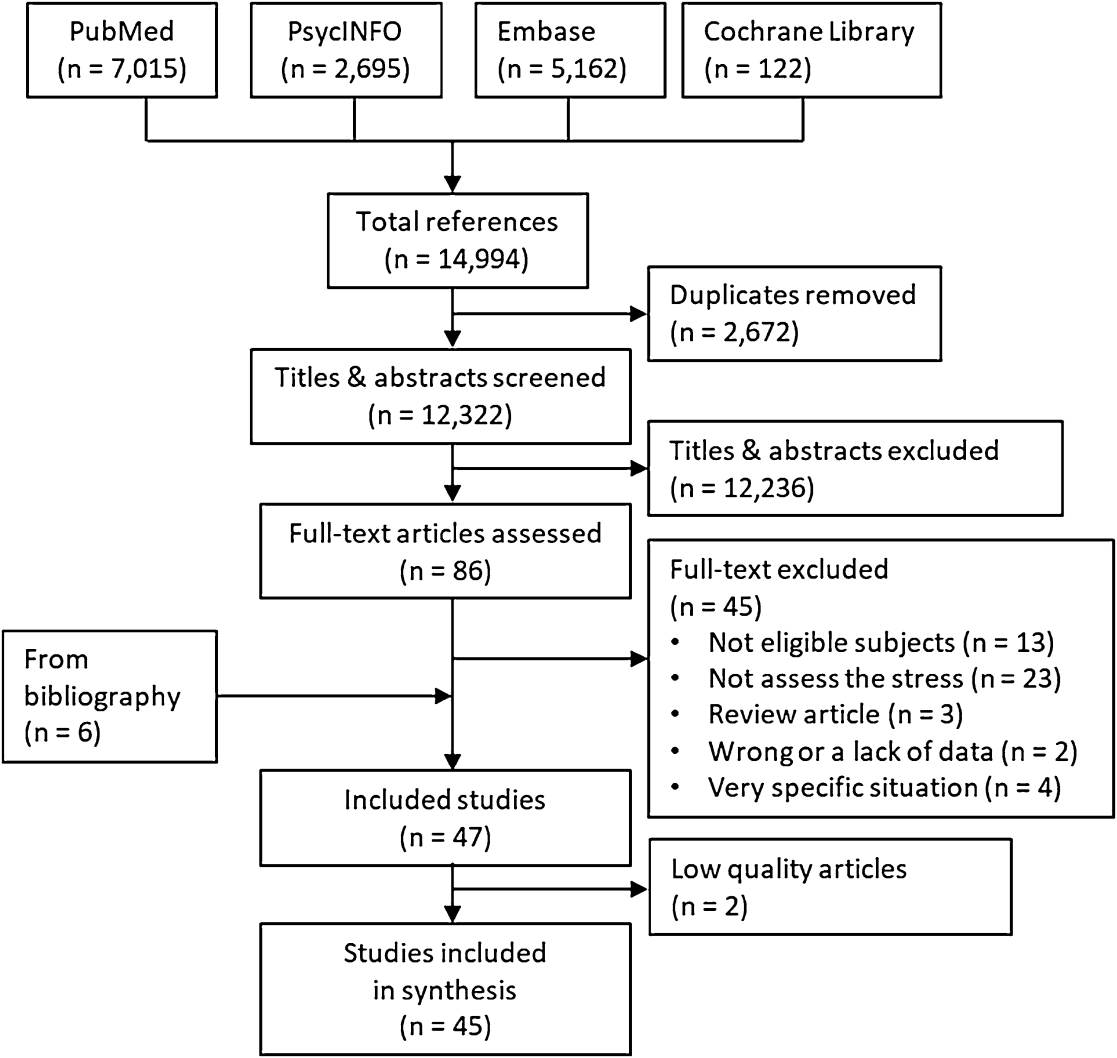
Flow diagram of included studies

In total, 14,994 articles from the four databases were searched (Fig. 1). Next, duplicates, papers not written in English, conference papers, review articles, editorials, commentaries and dissertations were omitted. Titles and abstracts of 12,322 articles were assessed, and then the full texts of 86 articles were collected. From them, 45 articles were excluded, and finally 6 articles were added from the references of included articles. In total, 47 articles were included to assess their quality.

### Study designs

Seven studies used a qualitative method; 33 studies used a quantitative method; 7 studies used a mixed model method. Among all the 47 retrieved studies, there was only 1 RCT. The assessments of risk bias or quality are shown in supplementary B, C, and D. One low-quality study in the part of qualitative study (Espino et al. 2002) and two studies with very high risk of bias (Deosthalee 2002; Zahid et al. 2003) were removed from the analysis. Finally, 45 studies were included in this review. The characteristics of the included studies are shown in Table 2.

**Table 2:** Study characteristics

Study	Study design	population	Sample size	Countries of origin	Host countries	Outcome measures	Results (stress factors)
Alderete et al. (2001)	Cross-sectional study	Mexican migrant farmworkers	1001	Mexico	USA	The risk of lifetime mood or anxiety disorders	Extension of their contact with the host society or becoming permanent settlers in the United States
Alkhadher and Al-Naser (2007)	Cross-sectional study	Teachers working in various American-system schools	77	USA	Kuwait	Occupational stress, occupational role, personal strain and personal resources	Locally hired teachers reported higher work role insufficiency than expatriate teachers
Al-Maskari et al. (2011)	Cross-sectional study	Immigrant workers [construction workers (40.8%), garage mechanics (2.0%), carpenters (21.7%) and others (35.5%)]	239	Indian (43.7%), Bangladeshi (9.2%), Pakistani (41.8%), non-national Arabs (2.8%) and others (2.5%)	UAE	The Depression Anxiety and Stress Scale (DASS-42)	Depression was associated with physical illness, working in the construction industry, earning less than 1000 UAE Dirham/month and working > 8 h/day. Workers with suicidal ideation were more likely to work < 8 h/day, earn less than 1000 Dirham/month and report an illness
Ayalon (2010)	Cross-sectional study	Filipino home care workers	178	Philippines	Israel	Intention to leave their work, depression and PTSD symptoms	Negative work/home experience was associated with depression and PTSD symptoms. Caring for elders with dementia was a risk factor of intention to leave their job
Bhanugopan and Fish (2006)	Cross-sectional study	Expatriate managers (21% respondents were working in manufacturing companies)	189	Australian (25.0%), New Zealander (15.0%), American (12.0%), British (5.0%), Asian (16.0%), Indian (7.0%), South Pacific Islander (6.0%)	Papua New Guinea	Job burnout	Role conflict, role ambiguity and role overload were related to job burnout. Role conflict was the main reason
Brown and James (2000)	Cross-sectional study	Filipino immigrant nurses or nurse’s aides	31	Philippines	USA	Blood pressure and catecholamine	The length of stay in the US was positively associated with the elevation of norepinephrine levels
Burkholder et al. (2010)	Cross-sectional study	Business travellers	12,942	USA	Multinational	International travellers’ objective and subjective health status	International travellers significantly associated with low BMI, low blood pressure, high consumption of alcohol, less sleeping hours, less confidence in keeping up with the pace of work and weakness of social ties with friends
Chen et al. (2010)	Cross-sectional study	Expatriates in a multinational company in the energy industry	556	31 countries	USA	Cross-cultural motivation, work adjustment and performance	Cross-cultural motivation was positively associated with work adjustment and job performance
Connor and Miller (2014)	Qualitative study	Filipino immigrant nurses	20	The Philippines	USA	The participant’s knowledge and perceptions of stress	Participants faced communication problems, discrimination, alienation and resettlement demands because of unexpected social and living environments
de Castro et al. (2010)	Cross-sectional study	Latino day labourers	30	Mexico (76.7%), Guatemala (10.0%), El Salvador (6.7%), Honduras (3.3%), Peru (3.3%)	USA	Work related, economic and social stressors using biological markers	There were no significant differences between objective health data and psychological burden score because of small sample size
Donlan and Lee (2010)	Cross-sectional study	Mexican migrant farmworkers	123	Mexico	USA	Depression scale	Culture-bound syndromes, poor or fair self-rated physical health, perceived language conflict and perceived discrimination were stress factors
Ea et al. (2008)	Cross-sectional study	Filipino nurses	96	Philippines	USA	Acculturation and job satisfaction	Age was negatively associated with job satisfaction
Espino et al. (2002)	Cross-sectional and qualitative study	Business travellers and their spouses of an American company	102	USA	Developing countries in Asia, Latin America, Africa and Eastern Europe	Stress of business travellers and their families	About 75% of the staff reported they felt high stress. The extent of stress was positively associated with the impact of their health and family
Fujishiro et al. (2010)	Cross-sectional study	Workers living in the USA who were born in foreign countries	915	Not mentioned	USA	Job control and demands, perceived job stress and self-rated health	Although job control was negatively associated with reporting fair/poor health among foreign-born English and Spanish users in the USA, it had no associations with job stress
Gabel et al. (2005)	Cross-sectional and qualitative study	Internationally assigned managers and their supervisors	39 for quantitative, 20 for qualitative study	Spain	Several Latin American countries	Emotional intelligence, job performance, satisfaction, cultural differences, perceived organisational support, cross-cultural training and spouse adjustment	Adjustment at the work place was associated with the intrapersonal emotional component
Griffin and Soskolne (2003)	Cross-sectional study	Thai migrant agricultural workers	221	Thailand	Israel	Psychological distress (depression and anxiety)	Migration stressors, the migrants’ traditional health beliefs, quality of current social relationships, drinking behaviour, younger age and occupational exposure were significantly associated with psychological distress
Grzywacz et al. (2010)	Cross-sectional study	Latino farmworkers	230	Mexico (94.8%)	USA	CES-D score for depression	Marital status, discrimination, the pace of work, crowded living conditions and concerns about documentation were the risk of depressive symptoms
Hayne et al. (2009)	Cross-sectional study	Nurses	15	Philippines	USA	Perception of their work environment, work satisfaction and indicators of job stress	Nurses were largely satisfied with their work. They reported that workload was appropriate, but 20% of nurses reported distress due to confusion about their role
Hiott et al. (2008)	Cross-sectional study	Latino migrant farmworkers	125	Mexico, Guatemala and Honduras	USA	The anxiety scale of the Personality Assessment Inventory, CES-D and alcohol dependence using the CAGE	Social isolation and working conditions were the risk factors of anxiety and depressive symptoms
Hoppe et al. (2009)	Case–control study	Latino warehouse workers	118	Latin America	USA	Job stress and psychological well-being	Fairness in management and support from supervisor were associated with their well-being
Hovey and Magana (2002)	Cross-sectional study	Mexican migrant farmworkers	65	Mexico	USA	The relationship between acculturative stress and anxiety	Elevated acculturative stress, low self-esteem, ineffective social support, lack of control and choice in living a migrant farmworker lifestyle, low religiosity, and high education were significantly related to high anxiety
Huang and Yang (2011)	Cross-sectional study	Foreign nurse aides who worked in long-term care facilities	71	Philippines, Indonesia and Vietnam	Taiwan	Work adjustment and work stressor	Patient care tasks were related to the foreign nurse aides’ personal relationships at work and their attitude to work. Work stressors were associated with work adjustment
Jassawalla et al. (2004)	Qualitative study	Managers working in the USA who experienced expatriate	8	Seven managers were from the USA, one manager was from the UK	Managers from the USA went to Brazil, Canada, Germany, Hong Kong, Japan, Switzerland, the UK and Venezuela. A manager from the UK went to the USA	Key challenges and cultural differences of the expatriates. Helpful personal strengths. Training for expatriate. Desirable preparation	The difficulty in the cross-cultural interpersonal conflict was a risk factor of stress
Karkar et al. (2015)	Cross-sectional study	Haemodialysis nurses	93	Philippines, India, Indonesia and Pakistan	Saudi Arabia	The amount of burnout and the impact of stress	Job insecurity was a risk factor of stress
Kawai and Mohr (2015)	Cross-sectional study	Japanese expatriate managers	125	Japan	Germany	Job satisfaction, task performance and work adjustment	Role ambiguity was negatively associated with job satisfaction and work adjustment
Lee et al. (2012)	Cross-sectional study	Korean–Chinese migrant workers (service workers 59.4%, construction workers 20.0%, and factory workers 15.9%)	170	China	Korea	job demands, insufficient job control, interpersonal conflict measures from the Korean Occupational Stress Scale and the CES-D	Acculturative stress, job demands, insufficient job control and interpersonal conflict were associated with depression
Lee et al. (2014)	RCT	Korean–Chinese female migrant workers	59	China	Korea	Musculoskeletal fitness, musculoskeletal symptoms, and acculturative stress	Intervention was stretching exercise + mobile phone text messaging and telephone counselling to increase self-efficacy and provide social support. Control was Stretching exercise only. Flexibility increased in both groups, but acculturative stress decreased only in the control group
Luxon and Peelo (2009)	Qualitative	International faculty in the UK higher education institutes	32	Sudan, China, Finland, etc. Not all countries were mentioned	UK	Development and assessment of English course for non-UK teachers	Teaching cultural surroundings, lack of information of daily life were the risk factors of stress
Negi (2012)	Cross-sectional and qualitative study	Latino day labourers	150	Immigrants born in Mexico (68%), Central America (31%) and the USA (< 1%)	USA	The association between discrimination, social isolation and factors of mitigating stress related to psychological distress	Discrimination and social isolation predicted psychological distress. Acculturation, religiosity, age and remittance were not significantly associated with psychological distress
Nilvarangkul et al. (2010)	Qualitative study	Laotian migrant workers in several different employment locations	70	Laos	Thailand	perception of stress	Living with poverty, non-standard wages and having limited choices, loneliness, abuse by employers and local people, distrusting their spouses, competition in the workplace and job uncertainty were risk factors of stress
Okamoto and Teo (2011)	Qualitative study	White-collar workers working for Japanese companies	37	Japan	Australia	Role stress (comprising role ambiguity and role conflict)	Insufficient competence in English, information shortage, differences in communication style and cross-cultural understanding were risk factors of stress
Pasca and Wagner (2012)	Case–control study	Professionals working in the fields of education, health care, and/or social work	84	Caucasian 54.8%, Hispanic 9.5%, Hispanic 9.5%, Other 26.2%	Canada	Satisfaction, mental health symptoms, relationship satisfaction, job satisfaction and occupational stress	Non-Canadian-born workers reported experiencing a higher level of somatic distress and paranoid ideation than Canadian-born
Rosenbusch et al. (2015)	Cross-sectional and qualitative study	The expatriates of overseas assignment in the last 3 years	111	25 nationalities (USA 14%)	27 countries (Switzerland 25% and USA 24%)	Cross-cultural adjustment	Spiritual, occupational and support stressors were associated with expatriate adjustment
Shaffer et al. (2013)	Cross-sectional study	Expatriates from nine nationalities living and working in Hong Kong	78	Nine nationalities	China (Hong Kong)	Expatriate pay satisfaction	Equity perceptions and appropriate assignment were positively associated with expatriate pay satisfaction
Silbiger and Pines (2014)	Cross-sectional study	Israeli expatriates	233	Israel	32 countries (USA 38% and UK 6.5%)	Adjustment, perceived stress level, burnout, work importance and withdrawal cognitions	Work importance was negatively correlated with burnout and positively correlated with stress
Snipes et al. (2007)	Qualitative study	Mexican immigrant farmworkers	69	Mexico	USA	The concept of stress	Language, traditional household duties for women, lack of having a consistent job, low income, injustice, family illness and the laws in the USA were risk factors for stress
Stahl and Caligiuri (2005)	Cross-sectional and qualitative study	The expatriates of German companies	116	Germany	Japan and USA	Work adjustment, interaction adjustment and intention to remain on the international assignment	The work adjustment was negatively associated with the combination of high position level and problem-focussed coping strategies
Stroppa and Spies (2011)	Cross-sectional study	White-collar employees on foreign assignment in small and medium-sized companies	127	Germany	China, USA, UAE, UK, Kazakhstan, Japan, India, Slovakia and others	Job stress and job satisfaction	Job stress was negatively associated with personal initiative and support from supervisors, but not associated with support from co-workers
Tsai and Salazar (2007)	Qualitative study	Chinese immigrants working for restaurants	18	China, Hong Kong and Taiwan	USA	physical, biological, enviro-mechanical, chemical, and psychosocial hazards	Workload, the hierarchical worker structure and communicating with customers in English were risk factors of stress
Tsutsumi et al. (2005)	Cross-sectional study	Workers at an electrical equipment manufacturing company	2233	Japan	USA, Brazil, France, Germany, Korea, UK, China, Iraq, Singapore and Ukraine	SDS, Sheehan’s Patient Rated Anxiety Scale and The Job Content Questionnaire	There were no significant differences in the SDS and Sheehan score between experienced and non-experienced group
Wadsworth et al. (2006)	Cross-sectional study	Workers in the UK	626	Black African–Caribbean and Bangladeshi	UK	Work related stress	Racial discrimination at work, gender, negative affect, contract, background noise and the work characteristics, effort–reward imbalance and job demands were associated with work stress
Wang and Kanungo (2004)	Cross-sectional study	Expatriates from multinational corporations	166	Taiwan, Hong Kong, Japan, India, Korea, North America and Europe	China (Beijing, Shanghai, Dalian and Suzhou)	Psychological well-being	Overseas experience and establishing the social network were related to psychological well-being
Wang and Takeuchi (2007)	Longitudinal study	Expatriates from a multinational manufacturing company	183 (time 1), 148 (time 2)	USA 69.4%, Canada 14.8%, Australia 11.5%	China	Goal orientation, perceived organisational support, expatriate adjustment, premature return intentions and job performance	Avoiding goal orientation was positively associated with work stress, and perceived organisational support was negatively associated with work stress
Weishaar (2008)	Qualitative study	Polish migrants working in manual and low skilled jobs	17	Poland	Scotland, UK	Stressors and individual experiences of the health impact of acculturative stress	Everyone mentioned the communication problems in English, which were the barriers for addressing the information. Workers felt difficulty in registration, information about accommodation and work, taxation and benefits. Wage inequalities such as low wage and high workloads were the main factor of stress
Winkelman et al. (2013)	Cross-sectional and qualitative study	Latino farmworkers	29 for qualitative, 57 for quantitative study	Latin America	USA	Stress, depression, and coping behaviours	Family situations, work environment, documentation status and lack of resources were the risk factors of stress and depression

### Subjects

Subjects are office, manual, healthcare, educational and agricultural workers. About 30% of studies surveyed expatriates. Two studies surveyed business travellers who had stayed in the host country for a short term. Other studies were about immigrants or did not show the duration of stay in the host country. The host country in about half of the studies was the US, and the subjects of these ten studies were from Latin America.

### The duration of stay

There was a wide range of durations of stay, from less than 5 days to over 15 years.

The most frequent duration of business visits among surveyed workers at a US multinational corporation was < 5 days/trip at 1–5 trips/year (Burkholder et al. 2010). The average duration of stay among business travellers at another US multinational corporation was 86.5 days (Espino et al. 2002).

A study about expatriates suggested that the length of stay in the host country was not associated with non-work adjustment (Stahl and Caligiuri 2005). The survey of a German multi-site study also showed that the length of stay was not related to job satisfaction and stress (Stroppa and Spies 2011).

In terms of immigrants, consensus was not reached on the influence of the length of stay on work and acculturation stress. Studies suggested that permanent settlers among Mexican immigrants had better mental health (Alderete et al. 2001), and that the length of stay and acculturation had a positive correlation with job satisfaction (Ea et al. 2008). However, the length of stay in the US was positively associated with the elevation of norepinephrine levels among Filipino immigrant nurses or nurses’ aides (Brown and James 2000).

### Stressors

In the present study, the evidence level of each study was not high. Only one study was designed as an RCT and about one-third of the studies were qualitative. From the qualitative studies, the problems of foreign-born workers were reported, and then the six domains (communication, cultural differences in the workplace, daily life, relationships with family and colleagues, financial problems and social inequality) were decided.

#### Communication

Language was a strong barrier to communication, which made it hard to address the information (Weishaar 2008). Expatriates felt frustration when facing a new language and had difficulty making new friends (Rosenbusch et al. 2015; Okamoto and Teo 2011). Chinese immigrants working for US restaurants reported that talking to customers in English was stressful (Tsai and Salazar 2007). Filipino nurses in the US also reported difficulty in communicating in English (Connor and Miller 2014).

According to two quantitative studies, language was not related to job satisfaction and stress (Grzywacz et al. 2010; Stroppa and Spies 2011). The ability to understand English and the state of their visas were not related to migrant farmworkers’ stress (Grzywacz et al. 2010). However, perceived language conflict was one of the stress factors (Donlan and Lee 2010). Social isolation was also associated with psychological distress such as depressive and anxiety symptoms (Hiott et al. 2008; Griffin and Soskolne 2003). Establishing a social network was important to expatriates’ well-being (Wang and Kanungo 2004).

#### Cultural differences in the workplace

Expatriates did not have enough time to learn about their new environment, and the change increased their stress levels (Rosenbusch et al. 2015). Their understanding of the cultural differences in education was more important for newly assigned foreign-born teachers in the UK than English training (Luxon and Peelo 2009).

The motivation to understand cross-cultural differences was positively associated with work adjustment and job performance (Chen et al. 2010). Expatriates’ goal orientation was related to job performance (Wang and Takeuchi 2007). Perceived work importance was the mitigating factor in work stress (Silbiger and Pines 2014). Role conflict, role ambiguity and role overload were positively associated with job burnout and dissatisfaction (Bhanugopan and Fish 2006; Kawai and Mohr 2015). However, according to a study among teachers in Kuwait, locally hired teachers reported higher work role insufficiency than expatriate teachers (Alkhadher and Al-Naser 2007). Although job control was negatively associated with reporting fair/poor health among foreign-born English and Spanish users in the US, it had no associations with job stress (Fujishiro et al. 2010). In addition, high job demand and low job control affected the personal relationships and work motivations of foreign-born nurses’ aides in Taiwan (Huang and Yang 2011).

#### Daily life

The balance between meeting an expectation at work and also at home was difficult (Rosenbusch et al. 2015). A sense of losing their language and culture was a stress factor for farmworkers in the US (Snipes et al. 2007). Family situations, work environment, documentation status and lack of resources were also stress factors for farmworkers in the US (Winkelman et al. 2013). It was recommended that information on accommodation and finance should be provided to newly assigned foreign-born teachers in the UK (Luxon and Peelo 2009).

International business travel correlated with increased alcohol consumption, difficulty in sleeping and less confidence in dealing with their work (Burkholder et al. 2010). In a Canadian study, non-Canadian-born immigrant workers reported a higher level of physical and psychological symptoms than Canadian-born workers (Pasca and Wagner 2012). Acculturation stress was also related to psychological problems (Lee et al. 2012), while low control and low religiosity among immigrant farmworkers were associated with anxiety (Gabel et al. 2005).

#### Relationships with family and colleagues

Expatriates and their families felt that it was difficult to keep in touch with their friends (Rosenbusch et al. 2015). Conflicting with co-workers was stressful for expatriates. To prevent such stress, selecting managers and providing training to both managers and expatriates before their assignments were helpful (Jassawalla et al. 2004). Many expatriates reported that the role of spousal adjustment was important (Gabel et al. 2005).

About 75% of business travellers and their families reported that they felt high stress levels. The assignment also negatively affected their children’s behaviours (Espino et al. 2002). Expatriates’ personal initiative as derived from supervisors, but not from co-workers, was related to job satisfaction, stress and performance (Stroppa and Spies 2011). Although there was no significant difference in the depression and anxiety scale between experienced and inexperienced workers on overseas assignments, white-collar workers in the experienced group received more supervisor support than the non-experienced group (Tsutsumi et al. 2005). Organisational support and well-established nurse/doctor relationships were reported as being beneficial by Filipino nurses in the US (Hayne et al. 2009).

#### Financial problems

According to a study among expatriates living in Hong Kong, equity perception and appropriate assignments were positively associated with pay satisfaction (Shaffer et al. 2013). Job insecurity was the major stress factor for expatriate nurses, which caused burnout and frustration (Karkar et al. 2015).

Wage inequalities, such as low pay and high workloads, were the main factor in work-related stress (Weishaar 2008). The job skills of immigrant workers were low as a result of low education status, which affected their job opportunities and fostered low-income status (Hayne et al. 2009). Many Filipino immigrant nurses in the US send money to their families in their home country, which was a major stress factor because this remittance was deducted from their living expenses (Connor and Miller 2014). A study among immigrant workers in the UAE found that poor income (< 1000 UAE Dirham/month) was one of the risk factors for depression and suicidal ideation (Al-Maskari et al. 2011).

#### Social inequality

Non-standard wages, having limited choices and abuse from employers and local people were the factors in the perception of stress (Nilvarangkul et al. 2010).

Discrimination is often reported to be a stressor for foreign-born workers. Filipino immigrant nurses faced discrimination, bullying, alienation and resettlement problems in the process of adaptation to their new country (Connor and Miller 2014). Workers from less-developed countries tended to perceive discrimination from the host country, which can be a risk factor for depression (Negi 2012; Wadsworth et al. 2006; Ayalon 2010). African-Caribbean females who experienced racial discrimination tended to perceive this as part of work stress (Wadsworth et al. 2006). For Latino migrant workers, management fairness and supervisor support were strongly related to their well-being (Hoppe et al. 2009).

## Discussion

The results of the present study can be greatly helpful in understanding the stress structure of working abroad and because there are many foreign-born workers all over the world. Few studies have elucidated foreign-born workers’ stress structure; therefore, these findings in the present study can provide useful data. Because this structure is different from native workers’ structure, particular attention is needed to manage foreign-born workers. From the present systematic review, six components of foreign-born workers’ stress were extracted, which were communication, cultural differences in the workplace, daily life, relationships with family and colleagues, financial problems and social inequality. They can be thought of as the core components of the stress model.

In terms of workers’ stress, there are two types: work stress and non-work stress. According to the National Institute for Occupational Safety and Health (NIOSH), stressors such as physical workload, spending a long time at work, shift work and qualitative burden affect the risk of injury and illness; however, the range of the stress reactions depends on each workers’ stress reduction capacity, which relates to factors such as age, sex, character, competence, support from one’s boss and colleagues and familial factors (Hurrell and McLaney 1988). When the stress model is mentioned, non-work factors and stress reduction capacity should also be evaluated. In the present study, six components cover work stress such as communication and cultural differences in the workplace, non-work stress such as daily life and financial problems, and workers’ stress reduction capacity such as relationships with family and colleagues and social inequality.

The most remarkable outcome of this systematic review was that six components were extracted. There was no study systematically evaluating the stress of foreign-born workers; therefore, the findings were highly valuable in the area of workers’ acculturation studies. Black et al. (1991) suggested a model of acculturation, which has five factors (individual, job, organisational culture, organisational socialisation and non-work) that relate to the degree of adjustment. In contrast, there are six components (communication, cultural differences in the workplace, daily life, relationships with family and colleagues, financial problems and social inequality) extracted in the present study. Although most factors overlapped, Black’s model did not include financial problems and social inequality. These factors are very important when workers from a less-developed country are assessed. In addition, previous theories were theoretically well-structured and well-organised, but they were not systematically developed. Because the present study was based on a systematic review, the components might be more comprehensive than previous theories on this point.

From the qualitative studies, it was found that fluency in the host country’s language was an important factor in communication. Many participants complained about speaking the host country’s language. However, surprisingly, there was no significant difference in language competence seen in the results of quantitative studies. Speaking a host country’s language was not an important factor for foreign-born workers. Three reasons can be considered. First, workers may need to speak only when it is related to their job, and speaking their own language can be an advantage. Second, some assignments do not require employees (e.g. farm workers) to speak the host country’s language. In addition, when the foreign-born worker’s community is large, they can speak their own language. Third, workers who are reluctant to speak another country’s language might not even go abroad. The important factor in the communication domain might be how workers are involved in the host country’s community rather than the issue of language proficiency. Social relations were one of the mitigating factors in stress for international assignees (Gabel et al. 2005).

When it comes to occupational training, to prevent work acculturation failure, although the present study could not show evidence on whether the training should be done before or after workers arrive in the host country, the contents of training should include both daily life and business acculturation. Harrison and Hopkins (1967) developed the first design for cross-cultural training using organisational structure and a problem-solving strategy. From the 25-year review, six approaches were suggested: cultural awareness, interaction, language, didacticism and experiential training (Littrell et al. 2006).

Support from family and co-workers is important in mitigating foreign-born workers’ stress levels. As part of the demand-control-support model, support is one of the most important factors in managing stress. The Health and Safety Executive also mentioned the importance of support when workers manage their stress (Health and Safety Executive 2005). There are many support resources for native-born workers, but there is a lack of support for foreign-born workers because they have moved to a new place. They need to participate in their new community and in social activities when they seek social support.

### Strengths and limitations

The strength of the present study is that both work and acculturation stress were evaluated. This made it possible to systematically assess what the stress of working abroad was and its relevant factors. Few evidence-based studies have evaluated both work and acculturation stress; therefore, the present study is useful if we want to understand the structure of foreign-born workers’ stress levels. The structure is similar to previous theories of acculturation, which supports the present study. In addition, the authors found two new risk factors, financial problems and discrimination, which were not mentioned in the previous theories. On the basis of the results of the present study, managers of foreign-born workers and workers themselves can ascertain what factors affect stress using the six extracted domains.

There are some limitations in the present study. First, this systematic review does not include enough evidence-based studies. Although, in the protocol, a GRADE evidence profile (GRADE Working Group 2004) and a meta-analysis were planned to be conducted, these could not be done because there was only one RCT. Furthermore, about one-third of the included studies were qualitative, which means there was a lack of deductive studies. Although the present study could propose the stress model, more experimental studies are needed to evaluate the model. Second, strong heterogeneity existed in the assessment method of stress among the included studies. The heterogeneity might be too inadequate to integrate the studies into a theory, so a new assessment tool needs to be developed. There are many tools for assessing acculturation, but few have been developed to assess acculturation related to work. Third, it is difficult to differentiate work stress and acculturation stress. Although there are many overlapping areas, stress of working abroad includes job stress; therefore, precise assessment of job stress is also needed. Finally, the search was conducted in November 2015. In 2016 and 2017, new studies reported that the supervisor’s support is related to the vigour of migrant workers (Hoppe et al. 2017), migrant nurses experienced work role and culture differences (Zhong et al. 2017), and the state of qualifications was the main factor influencing mental state (Sato et al. 2016). In several years, this systematic review will need to be updated.

## Conclusion

As international assignments and the number of immigrants increase, the importance of stress management among foreign-born workers also increases. There are many theories and assessment tools for acculturation, whereas theories and assessment tools for foreign-born workers’ stress do not exist. Since foreign-born workers are suffering from acculturation and occupational stress, establishment of a model and development of a questionnaire are needed. To establish the model of foreign-born workers’ stress, the present study was conducted. From the study, six components of foreign-born workers’ stress were extracted.

## Electronic supplementary material

Below is the link to the electronic supplementary material.

Supplementary material 1 (PDF 381 KB)
