# Supplementary material for: Stress of working abroad: a systematic review
Source: Int Arch Occup Environ Health. 2018 Jul 2;91(7):767–84. doi: 10.1007/s00420-018-1333-4 (PMC6132646; doi:10.1007/s00420-018-1333-4)
Supplement: Supplementary file 1 — Supplementary material 1 (PDF 381 KB) [file 420_2018_1333_MOESM1_ESM.pdf]

Supplementary

Supplementary A

Searching strategy

Search terms

PubMed:

((("work"[MeSH Terms] OR "work"[All Fields]) OR International[All Fields] OR Abroad[All Fields] OR ("emigrants and immigrants"[MeSH Terms] OR ("emigrants"[All Fields] AND "immigrants"[All Fields]) OR "emigrants and immigrants"[All Fields] OR "immigrant"[All Fields])) AND (("ethnology"[Subheading] OR "ethnology"[All Fields] OR "culture"[All Fields] OR "culture"[MeSH Terms]) OR ("social adjustment"[MeSH Terms] OR ("social"[All Fields] AND "adjustment"[All Fields]) OR "social adjustment"[All Fields] OR "adjustment"[All Fields] OR "adaptation, psychological"[MeSH Terms] OR ("adaptation"[All Fields] AND "psychological"[All Fields]) OR "psychological adaptation"[All Fields])) AND (("Stress"[Journal] OR "stress"[All Fields]) OR (mental[All Fields] AND ("disease"[MeSH Terms] OR "disease"[All Fields] OR "disorder"[All Fields]))))

PsychINFO:

((work or international or abroad or immigrant) and (culture or adjustment) and (stress or (mental and disorder))).mp. [mp=title, abstract, heading word, table of contents, key concepts, original title, tests & measures]

Embase:

((work or international or abroad or immigrant) and (culture or adjustment) and (stress or (mental and disorder))).mp. [mp=title, abstract, heading word, drug trade name, original title, device manufacturer, drug manufacturer, device trade name, keyword]

Cochrane Central Register of Controlled Trials (CENTRAL):

'(Work OR International OR Abroad OR Immigrant) AND (Culture OR Adjustment) AND (stress OR (mental AND disorder)) in Title, Abstract, Keywords, Publication Year from 2000 to 2015 in Trials'

Supplementary B

The quality assessment of qualitative studies

Study	1	2	3	4	5	6	7	8	9	10
Connor et al., 2014	Y	Y	Y	Y	Y	Y	Y	Y	Y	Y
Espino et al., 2002*	Y	N	-	-	-	-	-	-	-	-
Gabel et al., 2005*	Y	Y	Y	Y	N	N	Y	Y	Y	Y
Jassawalla et al., 2004	Y	Y	Y	Y	N	C	C	Y	Y	Y
Luxon et al., 2009	Y	Y	Y	Y	Y	Y	C	Y	Y	Y
Negi, 2012*	Y	Y	Y	Y	Y	Y	Y	Y	Y	Y
Nilvarangkul et al., 2010	Y	Y	Y	Y	Y	C	Y	Y	Y	Y
Okamoto et al., 2011	Y	Y	Y	Y	Y	C	C	Y	Y	Y
Rosenbusch et al., 2015*	Y	Y	Y	Y	Y	C	C	Y	Y	Y
Snipes et al., 2007*	Y	Y	Y	Y	Y	Y	C	Y	Y	Y
Stahl et al., 2005*	Y	Y	Y	Y	Y	C	C	Y	Y	Y
Tsai et al., 2007	Y	Y	Y	Y	Y	C	C	Y	Y	Y
Weishaar, 2008	Y	Y	Y	Y	Y	C	Y	Y	Y	Y
Winkelman et al., 2013*	Y	Y	Y	Y	Y	C	N	Y	Y	Y

1. Was there a clear statement of the aims of the research?

2. Is a qualitative methodology appropriate?

3. Was the research design appropriate to address the aims of the research?

4. Was the recruitment strategy appropriate to the aims of the research?

5. Was the data collected in a way that addressed the research issue?

6. Has the relationship between researcher and participants been adequately considered?

7. Have ethical issues been taken into consideration?

8. Was the data analysis sufficiently rigorous?

9. Is there a clear statement of findings?

10. How valuable is the research?

Y, Yes; C, Can't tell; N, No.

*; mixed model study.

Supplementary C

The risk of bias assessment for non-randomised studies

Study	Domain					
	1	2	3	4	5	6
Case-control study design						
Wang et al., 2007	Low	Low	High	High	Low	Unclear
Cohort study design						
Hoppe et al., 2009	Low	Low	High	High	High	Unclear
Pasca et al., 2012	Low	Low	High	High	Low	Unclear
Cross-sectional study design						
Alderete et al., 2001	Low	Low	Low	High	Low	Unclear
Alkhadher et al., 2007	Low	High	High	High	High	Unclear
Al-Maskari et al., 2011	Low	Low	High	High	High	Unclear
Ayalon, 2010	High	Low	High	High	Unclear	Unclear
Bhanugopan et al., 2006	Low	Low	High	High	High	Unclear
Brown et al., 2000	Low	Low	High	High	Low	Unclear
Burkholder et al., 2010	Low	Low	High	High	Low	Unclear
Chen et al., 2010	Low	Low	High	High	Low	Unclear
de Castro et al., 2010	Low	High	High	High	Low	Unclear
Deosthalee, 2002	High	High	High	High	Unclear	Unclear
Donlan et al., 2010	Low	High	High	High	High	Unclear
Ea et al., 2008	Low	High	High	High	Low	Unclear
Espino et al., 2002*	Low	High	High	High	High	Unclear
Fujishiro et al., 2010	Low	Low	High	High	High	High
Gabel et al., 2005*	Low	High	High	High	High	Unclear
Griffin et al., 2003	Low	Low	High	High	Low	Unclear
Grzywacz et al., 2010	Low	Low	High	High	Low	Unclear
Hayne et al., 2009	Low	High	High	High	High	Unclear
Hiott et al., 2008	Low	Low	High	High	Low	Unclear
Hovey et al., 2002	Low	Low	High	High	Unclear	Unclear
Huang et al., 2011	Low	High	High	High	Low	Unclear
Karkar et al., 2015	Low	High	High	High	Unclear	Unclear
Kawai et al., 2015	Low	Low	High	High	High	Unclear
Lee et al., 2012	Low	Low	High	High	Low	Unclear

Negi, 2012*	Low	Low	High	High	Unclear	Unclear
Rosenbusch et al., 2015*	Low	Low	High	High	Unclear	Unclear
Shaffer et al., 2013	Low	Low	High	High	High	Unclear
Silbiger et al., 2014	Low	Low	High	High	High	Unclear
Snipes et al., 2007*	Low	High	High	High	Unclear	Unclear
Stahl et al., 2005*	Low	Low	High	High	Unclear	Unclear
Stroppa et al., 2011	Low	Low	High	High	Low	Unclear
Tsutsumi et al., 2005	Low	High	High	High	Low	Unclear
Wadsworth et al., 2006	Low	Low	High	High	Low	Unclear
Wang et al., 2004	Low	Low	High	High	Low	Unclear
Winkelman et al., 2013*	Low	High	High	High	Unclear	Unclear
Zahid et al., 2003	High	High	High	High	Unclear	Unclear

1. The selection of participants
2. Confounding variables
3. Measurement of exposure
4. Blinding of outcome assessments
5. Incomplete outcome data
6. Selective outcome reporting

Supplementary D

The risk of bias assessment for a randomised controlled trial

Study	Domain						
	1	2	3	4	5	6	7
Lee et al., 2014	High	Unclear	Unclear	Low	Low	Unclear	High

1. Random sequence generation
2. Allocation concealment
3. Blinding of participants and personnel
4. Blinding of outcome assessment
5. Incomplete outcome data
6. Selective reporting
7. Other bias
